# Prospects on Tuning Bioactive and Antimicrobial Denture Base Resin Materials: A Narrative Review

**DOI:** 10.3390/polym15010054

**Published:** 2022-12-23

**Authors:** Yousif A. Al-Dulaijan, Abdulrahman A. Balhaddad

**Affiliations:** 1 Department of Substitutive Dental Sciences, College of Dentistry, Imam Abdulrahman Bin Faisal University, P.O. Box 1982, Dammam 31441, Saudi Arabia; 2 Department of Restorative Dental Sciences, College of Dentistry, Imam Abdulrahman Bin Faisal University, P.O. Box 1982, Dammam 31441, Saudi Arabia

**Keywords:** *Candida albicans*, biofilms, quaternary ammonium compounds, stomatitis

## Abstract

Denture base resin (DBR) materials are used in dentistry in constructing removable dentures and implant-supported prostheses. A plethora of evidence has demonstrated that DBR materials are associated with a high risk of denture stomatitis, a clinical complication where the soft oral tissues underneath the resin-based material are inflamed. The prevalence of denture stomatitis among denture wearers is high worldwide. Plaque accumulation and the infiltration of oral microbes into DBRs are among the main risk factors for denture stomatitis. The attachment of fungal species, mainly *Candida albicans*, to DBRs can irritate the underneath soft tissues, leading to the onset of the disease. As a result, several attempts were achieved to functionalize antimicrobial compounds and particles into DBRs to prevent microbial attachment. This review article explored the advanced approaches in designing bioactive and antimicrobial DBR materials. It was reported that using monomer mixtures, quaternary ammonium compounds (QACs), and organic and inorganic particles can suppress the growth of denture stomatitis-related pathogens. This paper also highlighted the importance of characterizing bioactive DBRs to be mechanically and physically sustainable. Future directions may implement a clinical translational model to attempt these materials inside the oral cavity.

## 1. Introduction

Edentulism, partial or complete, is an irreversible oral condition characterized by full or partial loss of teeth due to dental caries or periodontal diseases. While the prevalence of tooth loss has compacted over the last decade, edentulism remains a key global burden, extremely among geriatric patients [1]. Globally, the prevalence of complete edentulism can be ranged from 1.3% up to 78% for individuals over 65 years [2]. Despite the booming of implant dentistry, the use of removable dentures is still an effective prosthetic treatment option for the continuation of a healthy lifestyle through restoring patients’ function and esthetics [3]. 

In previous years, numerous materials were used for the fabrication of denture base resins (DBRs). These materials include wood, bone, ivory, porcelain, gold, vulcanite, tortoiseshell, and gutta-percha. Nevertheless, these materials have one or more characteristic disadvantages such as warped, unstable, lack of esthetic, difficulty of adjustment and modification, and hygienic concenters [4]. Therefore, to overcome these drawbacks, several efforts were conducted to develop desirable materials such as dental acrylic resins. 

In 1936, acrylic polymers like poly methyl methacrylate (PMMA) were first introduced in sheet form by Rohm and Hass company [5] and one year later in powder form by Nemours [4]. In the same year, Dr. Walter Wright popularized the use of PMMA for DBRs, making it the major polymer used for the fabrication of dental prostheses [5]. 

There are numerous advantages of PMMA, including the simplicity of processing and manipulation, functional stability, and reliability in the oral environment, in addition to the lighter weight, relatively cheaper, and satisfactory aesthetics [6,7]. Other PMMA features to highlight are superior biocompatibility, lack of odor and taste, absence of tissue irritation and toxicity, with the ability to adhere to the acrylic teeth chemically [8,9]. 

Despite the numerous advantages of PMMA, the nature of this material can render them vulnerable to biofilm adhesion and growth, leading to the development of oral infections such as denture stomatitis (DS), a common clinical condition affecting denture wearers, which is described as inflammation and erythema of the oral mucosal of denture bearing areas. Several reports have revealed that DS affects up to two-thirds or more of denture wearers [10,11,12,13]. Despite its commonality, the exact cause of the disease is still unknown, considering that it is a multifactorial inflammatory condition. However, three possible causes of DS were reported in the literature: trauma, allergic reaction, and microbial infection. 

Trauma is one of the potential causes. In a study by Nyquist, it was shown that DS was associated with ill-fitting dentures or with occlusal trauma [14]. There were several articles in agreement with Nyquist’s findings [15,16]. In addition, it was reported that localized mild lesions of DS could be resolved by trauma management [14].

Past publications suggested another possible cause of DS, which is contact allergy. Contact allergy may result from leaching out of uncured monomer [17,18]. Therefore, it would be predictable that an allergic reaction would start immediately, which is not the case with DS. Hence DS is commonly associated with old and neglected dentures [19]. Another study reported that the use of modern denture materials had rendered the allergic reaction, eliminating it as a possible cause of DS [20]. 

It was illustrated that the most common cause of DS is related to microbial infections, dominated mainly by fungal species, and referred to as oral candidiasis [21]. Several studies reported the risk factors associated with DS and oral candidiasis such as wearing complete denture versus partial prosthesis [22]; having a maxillary denture against wearing a mandibular denture [23]; female gender in contrast to male [23]; poor denture maintenance and care [24]; using denture during the night [25]; ill-fitting denture [26]; tobacco consumption [25]; diabetes mellitus [27]; prolonged use of antibiotic [28]; immune deficiencies [28]; nutrition deficiencies [29]; and altered salivary secretions [30]. 

**Figure 1:**
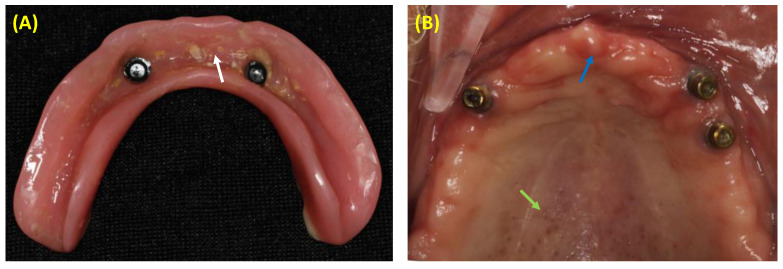
Clinical photos: (**A**) Mandibular overdenture with biofilm accumulation (white arrow), which could be associated with the onset of denture stomatitis; (**B**) Edentulous maxilla representing the clinical features of denture stomatitis such as pinpoint inflammation of minor salivary glands (green arrow), and diffuse erythema of the anterior alveolar ridge (blue arrow).

Clinically, DS is commonly asymptomatic; only a few denture wearers will experience itching, pain, or burning sensation [31]. Moreover, DS can be diagnosed clinically as the presence of inflammation or swelling of mucosal tissues beneath the denture [20]. As regards the clinical presentation, it can be categorized based on Newton’s classification as pinpoint hyperemia and inflammation, diffuse erythema, or granular inflammation (Figure 1) [32].

*Candida albicans* is considered one of the microorganisms presented in a healthy oral environment and is highly associated with the onset of DS. As a result of the microbial imbalance, *C. albicans* underneath the denture base may infiltrate and denture surface, establish microbial biofilms, and lead to the formation of mucosal inflammations. These lesions may cause oral infection and periodontal disease [33,34]. Other than localized conditions, in chronic denture wearers and immunocompromised patients, it was found that the denture microorganism can be associated with systemic diseases such as infectious endocarditis, aspiration pneumonia, respiratory candidiasis, gastrointestinal infections, and even death [35,36].

Although the mechanism of microbial adhesion to DBR is not yet well-known, the association between DBR surface roughness, salivary pellicles, and hydrophobic or electrostatic bonds is believed to be a possible rationalization [37]. Furthermore, the irregularities of the fitting (intaglio) surface of the denture base act as a reservoir for biofilm to attach to the palatal mucosa, providing a suitable environment for accumulation and pathogenesis [38]. Besides, since the material is inadequate in all aspects, the mechanical properties may decay during the clinical service, resulting in inferior surface characterizations and uncertain mechanical properties, such as impact strength, hardness, fracture, and flexural strength [39,40,41]. Such deterioration in these properties may accelerate the development of irregularity over the DBRs surfaces, leading to more surface roughness and plaque accumulation [39]. 

Several studies reported the correlation between surface roughness and microbial accumulation, where higher surface roughness leads to greater biofilm adhesion [20,42,43,44]. This can be explained by two factors; (1) increased surface area and (2) the presence of depth irregularities that cannot be managed by regular cleaning forces [44]. Also, the hydrophobicity of DBRs was reported to be associated with microbial adhesion due to wettability and water adsorption [44,45]. 

**Figure 2:**
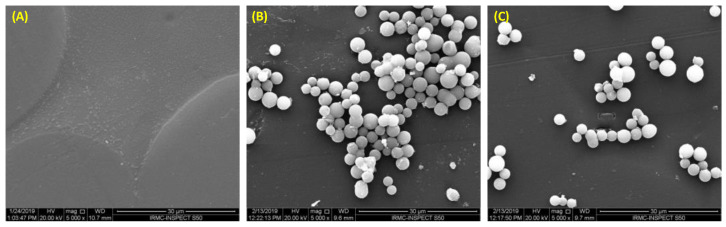
Scanning Electron Microscope (SEM) images of the black substrate and the microbial cells: (**A**) Denture base resin prior to biofilm challenge; (**B**) Candida adhesion to the conventional denture base resin; and (**C**) Candida adhesion on the antimicrobial denture base resin.

Considering these limitations observed over the use of DBRs, several efforts were conducted to inhibit the colonization of *C. albicans* biofilms on the DBRs by the addition of bioactive and antimicrobial agents to potentially prevent the development of denture stomatitis and to enhance their properties (Figure 2) [21,39,46]. In dentistry, imparting bioactivity into different restorative materials has been attempted to minimize the risk of different biofilm-triggered diseases, such as dental caries, endodontic infections, and periodontitis [47,48,49,50,51]. These materials include dental restorations [52,53,54], dental adhesives [55,56,57], cements [58], sealants [59], implant prostheses [60], and endodontic sealers [61]. Among them, DBRs have received significant concern due to the high prevalence of denture stomatitis [11,17,31]. Therefore, incorporating bioactive compounds and antimicrobial agents into DBRs. The purpose of this article is to review various aspects of antimicrobial agents that are incorporated into PMMA resin, as well as their performances when used for different dental applications. We focused on this review to include only the compounds and agents that were embedded within the PMMA material. Materials that were used as a coating agent were not discussed. All the articles in English without a specific timeline were extracted from PubMed and included in this review.

## 2. Methods Used for Tuning Bioactive and Antimicrobial DBR Materials

**Figure 3:**
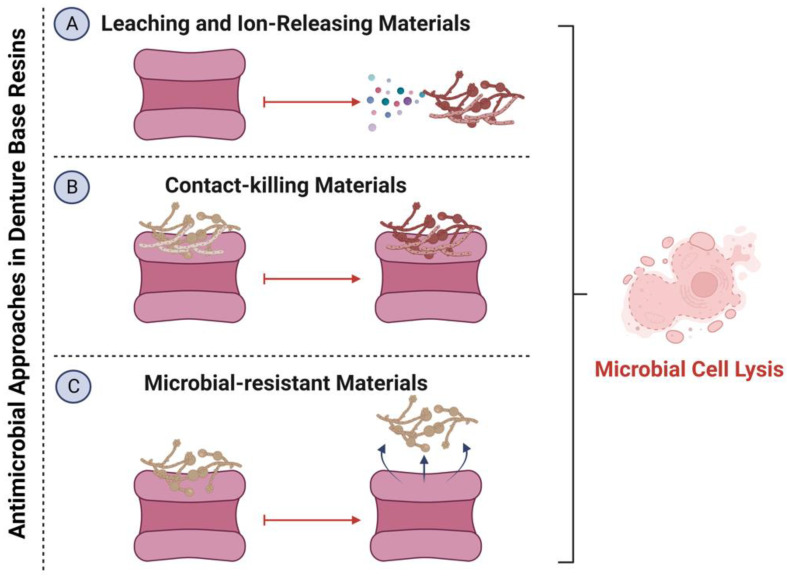
Several approaches have been investigated to improve the antimicrobial action of denture base resins: (A) the use of agents or ion releasing material that are capable of targeting the accumulated biofilms when they are released from the resin matrix system; (B) contact-killing materials that can eradicate the attached biofilms upon contact without release or leaching; and (C) microbial-resistant materials that can prevent the microbial attachment but without direct killing.

Several methods with different mechanisms have been investigated to impart bioactivity into DBRs (Figure 3) [21,39,46]. These methods can be classified into three categories: (1) the use of agents or ion releasing material that are capable of targeting the accumulated biofilms when they are released from the resin matrix system, (2) contact-killing materials that can eradicate the attached biofilms upon contact without release or leaching, and (3) microbial-resistant materials that can prevent the microbial attachment but without direct killing. The bioactive agents also could be classified according to their chemical nature into organic and inorganic materials, polymeric compounds, and antifungal medicaments, as shown in Table 1. The main purpose of such integration is to minimize microbial attachment and, therefore, prevent the onset of DS. 

### 2.1. The Incorporation of Organic Compounds into DBRs

Natural products are widely used as therapeutic agents. The main advantages of using natural products in drug delivery are the availability and the less likelihood of inducing microbial resistance [92]. Several investigations have utilized organic compounds to enhance the antimicrobial properties of DBRs, which are discussed below. 

#### 2.1.1. Henna

Henna, *Lawsonia inermis*, is a plant used to color skin and hair, utilizing the dye molecules inherited in this plant [93]. Henna has been featured with several biological and antimicrobial properties. It has been found effective against several viruses, fungi, and bacterial species [94]. In one dental study, heated cured acrylic used in denture construction was mixed with 1, 2.5, 5, 7.5, and 10 wt.% of Henna [62]. A dose-dependent effect was observed when the acrylic samples were exposed to *C. albicans* biofilm. Incorporating 1% of Henna reduced the *C. albicans* growth by 0.5-log. While the maximum amount of inhibition was observed when 7.5 and 10% of Henna were incorporated, as 1.5 and 2.5-log reduction, respectively, was achieved [62]. One of the limitations of this study is that the mechanical properties of the Henna-containing samples were not investigated. This is essential as bioactive resin-based materials must be mechanically stable to avoid any mechanical failure during their clinical service inside the oral cavity. In another investigation, this concern was resolved where the mechanical and antifungal properties of heat-polymerized acrylic resins containing 0.5, 1.0, 1.5, or 2.0 wt.% of white and natural Henna were investigated [63]. The flexural strength, surface roughness, and translucency values of Henna-containing DBRs were negatively affected by increasing the concentration of white and natural Henna. Only the 0.5 wt.% of white and natural Henna demonstrated good mechanical and physical properties compared to the control. Opposite to most of the antimicrobial compounds, increasing the Henna concentrations increased the *C. albicans* biofilms. Only 0.5 wt.% of white and natural Henna reduced the *C. albicans* significantly compared to the control with no addition [63]. This could be attributed to the increased surface roughness associated with the other concentrations, which could facilitate the attachment of *C. albicans*.

#### 2.1.2. Phytoncide Microcapsules 

One of the promising organic compounds derived from plants is phytoncide microcapsules. It has the capability to modify the human immune response by increasing the activities of natural killer cells [95]. Its incorporation into DBR as an antifungal approach was attempted in several investigations. Phytoncide microcapsules were incorporated into DBR in a range between 0.5–5 wt.% [96]. The flexural strength value was significantly reduced as the phytoncide microcapsules increased. Concentrations higher than 2% were associated with a flexural strength value lower than 60 MPa. Besides, more topography changes were observed over the DBR surface in the scanning electron microscopy with high concentrations. For the antibiofilm assay, 2.6% of phytoncide microcapsules was determined as the minimum inhibitory concentration against *C. albicans* before the incorporation into the DBR [96]. This study could be more valuable if the fungal biofilm accumulation over the DBR specimens was investigated. This was achieved in another investigation where different mass fractions, 1.25, 2.5, 3.75, and 5 wt.%, of phytoncide were incorporated into DBRs [64]. All the concentrations demonstrated excellent biocompatibility and fungal reduction against *C. albicans* grown over the synthesized materials. However, the 5% concentration was associated with a dramatic reduction in the flexural strength value [64]. In another investigation, two types of phytoncide microcapsules, A and B, were incorporated into 3D-printed DBR [65]. Increasing the concentrations of phytoncides A and B in the DBR did not affect the biocompatibility of the material. However, increasing the concentration was associated with increased surface roughness of the designed samples. For the antifungal properties, DBR containing 6 wt.% of type A and 15 wt.% of type B phytoncide microcapsules revealed a significant reduction against *C. albicans* growth [65]. The same authors in another study demonstrated that incorporating 6 wt.% of type A and 15 wt.% of type B phytoncide microcapsules into DBR was associated with a significant reduction in the flexural strength, elastic modulus, and microhardness of the DBR materials [66]. Such observations may suggest the need for more characterization of this compound to be functionalized into DBRs without compromising the mechanical properties.

#### 2.1.3. Neem

Neem is another plant-derived organic compound used for many years as a potential agent for anti-inflammatory and antimicrobial purposes [97]. Its antifungal effect has been demonstrated in several investigations [98,99]. Exposing the DBR samples to neem extract was found effective in minimizing the adherence of *C. albicans* [100]. In a recent investigation, neem powder was incorporated into heat and auto-polymerized DBR at 0.5, 1, 1.5, 2, and 2.5 wt.% [67]. Incorporating 2.5 wt.% of neem was associated with around 2-log reduction against *C. albicans* growth over the neem-containing DBR samples [67]. As it was observed in other compounds, it will be interesting to evaluate the impact of neem incorporation on the mechanical and physical properties of the DBRs. Besides, concentrations higher than 2.5 wt.% could be attempted.

#### 2.1.4. 1,4-Diazabicyclo[2.2.2]octane (DABCO) Derivatives

DABCO is an organic catalyst compound used in the polymerization reaction. Its derivatives were recognized with various antimicrobial properties [101]. In one investigation, different DABCO derivatives were incorporated into DBR. Among them, C2DC11MAF derivative was found effective in inhibiting *C. albicans* growth with minimum cytotoxicity against periodontal ligament cells and gingival fibroblasts [68]. The positive charge of this derivative may contribute to its antifungal action by interacting with the negatively charged microbial membrane. Future studies utilizing DABCO derivatives may consider assessing the mechanical and physical properties of the designed DBRs.

### 2.2. The Incorporation of Inorganic Particles into DBRs

**Figure 4:**
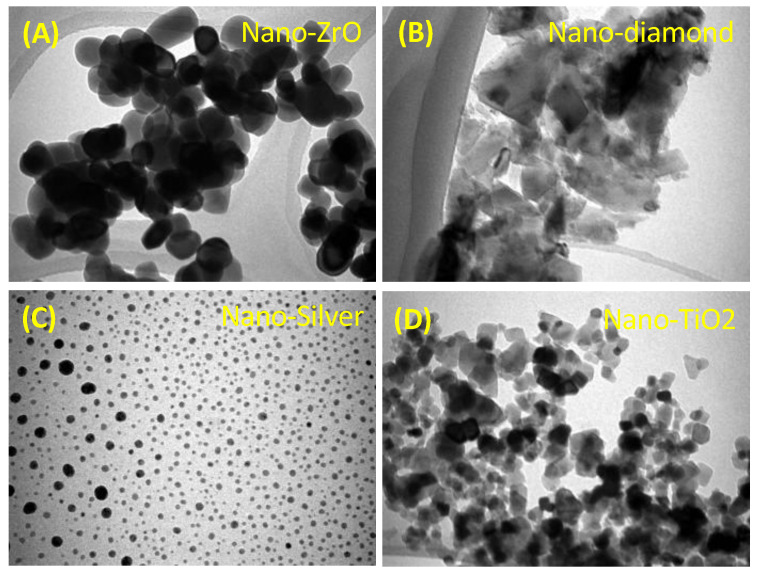
Transmission Electron Microscopy (TEM) images of different inorganic nanoparticles that could be incorporated into denture base resin to impart bioactivity and antimicrobial properties: (**A**) Nano-Zirconium Oxide; (**B**) Nano-diamond; (**C**) Nano-Silver; and (**D**) Nano-Titanium Dioxide nanoparticles.

Several studies have proposed the use of inorganic particles in dentistry to improve dental restorative materials’ mechanical and physical properties [49,50,102]. In the design of DBRs, many studies evaluated the incorporation of inorganic nanoparticles to impart bioactivity and minimize the onset of DS, such as nanodiamonds, nano-zirconium oxide, nano-silver, and nano-titanium dioxide **(Figure 4)**.

#### 2.2.1. Silver and Silver Zeolites 

The antimicrobial properties of silver micro and nanoparticles are well-known in the literature. The release of silver ions can attack the targeted microorganisms by several mechanisms, such as efflux pump alteration, membrane disruption, membrane permeability alteration, and leakage of intracellular contents [103]. The incorporation of silver particles into DBRs was heavily investigated. The initial investigations were conducted to improve the mechanical properties of DBRs [104,105]. Then, more studies were released to evaluate the antifungal properties of DBRs containing silver. In one of these studies, silver bromide/cationic polymer was incorporated into DBR at 0.1, 0.2, and 0.3 wt.% [69]. The mechanical properties of DBR containing silver bromide were not affected, as the flexural strength, elastic modulus, and microhardness values were within the normal range. Neither the topography characteristics nor the degree of conversion of the designed materials was compromised. The designed formulations demonstrated a significant reduction against *C. albicans* before and after one week of aging [69]. In another study, the incorporation of silver vanadate nanoparticles up to 10 wt.% was effective in eradicating the growth of *C. albicans* and *S. mutans*, without compromising the mechanical properties of the material [70]. The dose-dependent effect of silver nanoparticles loading was evident in one study, where the 72-h *C. albicans* biofilm adhesion was minimum at 5 wt.% loading compared to smaller concentrations [71]. In a recent interesting investigation, incorporating silver nanoparticles at the size of 20 nm improved the mechanical properties and the antifungal performance of DBRs [74]. Incorporating 0.5 to 1.5 wt.% of silver nanoparticles reduced the *C. albicans* biofilm by 1 to 1.5-log, with the maximum amount of reduction observed in the 1.5 wt.% group. One of the drawbacks observed in this study was the reduced strength and translucency of the material after containing the silver nanoparticles [74]. It is important to mention that the addition of silver also may negatively affect the color of such materials [102], which could impact the esthetic properties of the material to a certain extent. Future investigations may apply unique coating approaches to minimize the metallic color appearance of these particles and improve the esthetic of the designed materials.

#### 2.2.2. Pre-Reacted Glass Ionomer Fillers

The acid-base reaction of the pre-reacted glass ionomer fillers can release several ions, such as fluoride ions, to minimize the adhesion of microbes [106]. It has been illustrated previously that resin composite restorations containing pre-reacted glass ionomer fillers were associated with less accumulation of plaque [107]. Similarly, the incorporation of these fillers into DBR was attempted. The incorporation was achieved at three levels, 5, 10, and 20 wt.% [72]. Higher ion release from the fillers containing DBRs was observed. The attachment of the *C. albicans* was reduced significantly as the filler’s concentration increased. While the surface roughness was negatively affected by the filler’s incorporation, the surface roughness value was still lower than the critical value of 0.2 µm [72], a value that may induce plaque and biofilm accumulation. It would be more valuable to investigate this approach more comprehensively by evaluating other mechanical properties, such as flexural strength and contact angles. Besides, the rechargeability of the designed DBRs is worthy of being assessed, as this may assure long-term bioactivity induction of this material. These concerns were answered in another study where the strength and rechargeability of the glass containing DBRs were assessed [108]. It was found that incorporating glass fillers up to 20 wt.% can assure a flexural strength value of more than 65 MPa. It was also illustrated that several recharge cycles were achievable, assuring long-term ion release.

#### 2.2.3. Zinc Oxide (ZnO)

The antimicrobial properties of ZnO particles are attributed to their ability to induce oxidative stress damage against the cell membrane of the targeted cells [109]. As an antifungal strategy, ZnO nanoparticles were incorporated into DBRs at 1.25, 2.5, and 5 wt.% in two conditions, silanized and non-silanized [73]. DBRs with silanized ZnO nanoparticles demonstrated higher flexural strength values compared to their non-silanized counterparts. Similarly, the antifungal effect against *C. albicans* was more potent in the silanized groups. DBRs containing 5 wt.% of silanized particles reduced the growth by around 2-log compared to the control [73]. It was suggested that silanizing the ZnO nanoparticles into the coupling agent can maximize the surface-to-volume ratio via better maintaining the size of the particles and achieving better distribution homogeneity, resulting in a more antifungal effect [110]. It is worth saying that using small concentrations of less than 1 wt.% of ZnO nanoparticles may not induce any antifungal effect [111], mandating the need to use high concentrations to achieve the required antimicrobial action. 

#### 2.2.4. Zirconium Dioxide (ZrO_2_)

The use of ZrO_2_ in improving the physical characteristics and the antimicrobial properties of medical and dental materials is well evident in the literature [112]. The antifungal action of ZrO_2_ nanoparticles was observed via the ability of these particles to interfere with hyphae formation and the disruption of cell function, mainly by inducing oxidative stress [113]. In 2022, Gad et al. demonstrated the capabilities of ZrO_2_ nanoparticles at 0.5 to 1.5 wt.% to improve the flexural strength of DBRs by 10–25% compared to the parental formulation [74]. The same study revealed the ability of ZrO_2_-containing DBRs to inhibit the *C. albicans* biofilm growth by 0.5 to 1-log [74]. The ability of ZrO2-containing DBRs to induce an antifungal effect following the aging process was also demonstrated [75]. At 1, 2.5, and 5 wt.%, the amount of log reduction against *C. albicans* biofilms was the same, 0.5 to 2-log reduction, before and after 5000 cycles of thermocycling [75]. While the incorporation of ZrO2 nanoparticles is promising, it could be more implemented toward improving the strength of the DBR materials. A synergistic combination with a more potent antifungal agent may result in a sustained material with excellent mechanical and antifungal properties.

#### 2.2.5. Silicon Dioxide Nanoparticles (SiO_2_NPs)

SiO_2_NPs are one of the promising inorganic compounds to expand their uses in medicine and dentistry due to their excellent characteristics, such as improved surface adsorption and energy, homogeneous dispersion, and superior thermal resistance [114]. In dentistry, SiO_2_NPs can be used as optical modifiers and to improve the radiopacity of restorative materials [115]. As an approach to improve the mechanical and antifungal properties of DBRs, SiO_2_NPs were incorporated at 0.05, 0.25, 0.5, and 1 wt.% [76]. At low concentrations, SiO_2_NPs-containing DBRs accepted decreased contact angle and improved strength. However, with high concentrations, the surface roughness and translucency were negatively affected. A significant reduction against *C. albicans* biofilms was observed with increasing the concentration of SiO_2_NPs [76]. It is believed that the antimicrobial action of SiO_2_NPs is delivered by oxidative stress induction [116], which can damage the microbial membrane.

#### 2.2.6. Titanium Dioxide Nanoparticles (TiO_2_NPs)

**Figure 5:**
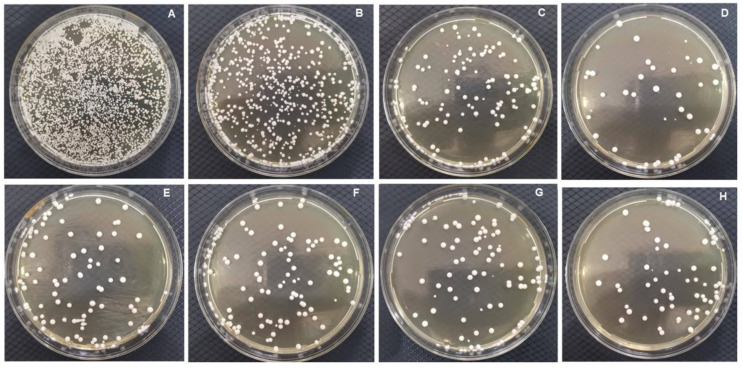
Candida count according TiO_2_ nanoparticle % and layering technique: (**A**) Negative; (**B**) control—Unmodified; (**C**) one-layer 1%; (**D**) one-layer- 2.5%; (**E**) double-layer 1%; (**F**) double-layer 2.5%; (**G**) dotted-layer 1% groups; and (**H**) dotted-layer 2.5% groups.

TiO_2_NPs demonstrate several advantages related to their high biocompatibility, chemical stability, and resistance to corrosion [39]. TiO_2_NPs can generate free radicals and singlet oxygen to target the membrane of different microbial species [117]. In one study, adding TiO_2_NPs to DBRs in different loading techniques at 1 and 2.5 wt.% reduced the growth of *C. albicans* by 0.5 to 1-log [77] **(Figure 5)**. It was found that adding TiO_2_NPs in the one-layer technique was associated with a significant reduction in the strength of DBRs. At the same time, packing the particles in two layers or dotted layer resulted in comparable flexural strength compared to the control [77]. Such packing techniques could be applied with other bioactive agents that may exert adverse effects on the mechanical properties of DBRs.

#### 2.2.7. Nanodiamonds (NDs)

NDs are highly biocompatible with wide-spectrum antimicrobial properties [78]. The oxygen-derived group within the chemical structure of nanodiamonds can interact with and disrupt the cell membrane of the targeted microorganisms [118]. It was found that adding 1 wt.% of nanodiamonds to DBRs inhibited the *C. albicans* growth by 1-log without compromising the mechanical properties of the material [119]. The antifungal effect of nanodiamonds was observed even with lower concentrations, such as 0.25 and 0.5 wt.% [79]. One of the main drawbacks of nanodiamonds incorporation is the reduced translucency of the constructed materials. More efforts could be directed to solve this issue by incorporating other coloring and optical modifiers into DBRs. 

### 2.3. The Incorporation of Polymeric, Quaternary Ammonium Compounds, and Protein-Repelling Agents into DBRs

Polymeric compounds have several uses in medicine and dentistry. These compounds have been used for decades to improve the function, delivery, and absorption of therapeutic agents [120]. In dentistry, polymeric compounds have been used in the design of different restorative materials, such as dental fillings, cements, adhesives, sealants, and DBRs [51,121]. Chitosan, as a natural polymeric material, has been well-recognized for its antimicrobial properties [122]. In one study, chitosan was incorporated into DBRs either directly via different mass fractions or by co-polymerizing the material with the methyl methacrylate monomer within the DBR material [80]. It was found that incorporating chitosan at 0.5–3 wt.% is effective in inhibiting the growth of *C. albicans* and *S. mutans* [80].

One of the recent potent antimicrobial quaternary ammoniums is dimethylaminohexadecyl methacrylate (DMAHDM). This compound has 16 alkyl chains, and it depends on the nitrogen-positive charge to interact and damage the cell membrane of the targeted microbes. DMAHDM has been extensively investigated, and it was found to impart bioactivity to different restorative materials such as dental fillings, cements, adhesives, and sealants. In DBRs, it was found that incorporating DMAHDM at 3 wt.% into DBR material reduced the growth of *C. albicans* by 1-log [86]. More reduction was observed when DMAHDM was combined with a protein-repellent agent named 2-methacryloyloxyethyl phosphorylcholine (MPC). However, DBRs containing DMAHDM with and without MPC were associated with significant concern due to the major reduction in the materials’ strength [86]. 

Minimizing the alkyl chain of DMAHDM from 16 to 14 resulted in another compound called dimethylaminododecyl methacrylate (DMADDM) [36,87]. Incorporating 5, 10, and 20 wt.% of DMADDM into DBRs did not affect the fracture strength, flexural strength, and surface roughness of the material [87]. At the same time, the metabolic activities and biofilms of *C. albicans* were significantly reduced in a dose-dependent manner [87]. DBRs containing DMADDM were also found effective in inhibiting multi-species biofilms [36]. Another QAM that was investigated is N,N-dimethylaminoethyl methacrylate (DMAEMA) [88]. It demonstrated a potent against *Escherichia coli*, *Staphylococcus aureus*, and *C. albicans* and achieved around 2-log reduction. However, the mechanical properties, such as flexural strength and water sorption, were severely compromised [88], suggesting the need for more characterization of this compound. Other compounds, such as methacryloyloxyundecylpyridinium bromide (MUPB) [123], fluoroalkyl acrylate [81], and 2-hydroxyethyl methacrylate ester [82], were also functionalized into DBRs to combat fungal growth established in vitro. One of the unique approaches to improve the mechanical and antifungal properties of conventional PMMA is the use of metal methacrylate monomers [83]. Incorporating zirconium methacrylate (ZM), tin methacrylate (TM), and di-n-butyldimethacrylate-tin (DNBMT) into PMMA was associated with good reduction against *C. albicans*. The degree of conversion, optical properties, and surface roughness of the modified PMMA were not affected. Besides, incorporating DNBMT, in particular, improved the hardness of DNBMT-containing DBRs [83]. 

A biocide polymer called poly (2-tert-butylaminoethyl) methacrylate (PTBAEMA) has been studied for its effect when incorporated into DBRs [84]. The addition of PTBAEMA to a heat-polymerized acrylic resin at 10% significantly reduced the growth of *S. aureus* and *S. mutans* by 4 and 2.5-log, respectively. However, this modification did not reduce the growth of *C. albicans* [84]. While this polymer did not affect the growth of *C. albicans*, an indirect effect on the fungal growth could be achieved by minimizing the growth of bacterial species, such as *S. mutans*, that can enhance the colonization and the adhesion of *C. albicans*. Another approach to combat fungal infections could be by modulating the oral environment to disfavor the growth of Candida. For example, phosphated poly(methyl methacrylate) polymer was found to reduce the adhesion of *C. albicans* by increasing the adsorption of histatin 5 in a phosphate density-dependent manner when it was incorporated at 15% into DBRs [85]. Such observations may allow discovering more approaches to minimize the fungal biofilm growth, not necessitating the direct antimicrobial effect against the targeted microorganisms. 

### 2.4. Antifungal Medicaments 

One of the strategies to impart bioactivity into DBRs is the incorporation of antifungal medicaments such as chlorohexidine and fluconazole, which are common dental disinfecting agents. These medicaments have been initially designed to disinfect and clean DBRs extra or intra-orally [124]. However, some researchers hypothesized that incorporating these materials in DBRs will result in a durable and long-lasting antimicrobial effect. In one study, chlorohexidine and fluconazole at 10 and 4.5%, respectively, were added [89]. It was found that adding fluconazole to PMMA did not affect the fracture toughness of the material, whereas Chlorohexidine addition negatively affected the fracture toughness [89]. Incorporating 10% of chlorhexidine into DBRs was found to inhibit the growth of *C. albicans* biofilms significantly [90]. In another investigation, chlorhexidine was incorporated into DBRs at 0.5, 1, and 2 wt.% [91]. A small amount of chlorhexidine release was detected for up to 28 days, with the maximum peak of release on the second day. Adding chlorhexidine did not affect the degree of conversion values of the designed specimens; however, the water sorption was increased among the chlorhexidine containing DBRs. For the antifungal properties, a larger inhibition zone was observed around the samples containing chlorhexidine compared to the control [91]. More investigations are needed to characterize the functionalization of chlorhexidine into DBRs. Besides, more attention is needed in regard to when the chlorhexidine release could be ineffective in preventing microorganisms’ adhesion.

## 3. Future Perspectives and Conclusion

As it is discussed in this review article, the incorporation of bioactive agents into DBRs has the potential to reduce the onset of DS. While the reported outcomes are promising, there are several aspects that require further clarification and evaluation. It is critical to observe that a considerable portion of the conducted investigations focused on the antimicrobial evaluation of the synthesized materials. While the mechanical, physical, and biological assessment of these materials was partially or completely neglected. It is important for DBR materials to be mechanically stable with good physical and biological properties. No matter how the material is potent against the oral microbes, inferior mechanical properties will accelerate the mechanical failure of the material inside the oral cavity, and the material will be highly subjected to fracture [50]. Inferior physical properties may also affect the esthetic appearance of the materials as well as the form and roughness [49]. Besides, materials with low biocompatibility may irritate the surrounding periodontium. Simply, designed materials with poor properties may lead to DS not by the lack of antimicrobial properties, but due to the topography changes that can facilitate microbial attachment [49]. 

Another aspect that was not addressed in most of the reported articles is the longevity of the antimicrobial action of the designed DBRs. Materials with potent antimicrobial action may demonstrate a decay in this property over time [125]. This is highly expected in leaching bioactive agents, where the bioactive agents will be released for a while, and then the amount of release will not be effective to prevent the onset of the disease. Such concern could be less important among contact-killing materials, where the material can co-polymerize with the resin matrix, and no leaching happens [125]. However, despite the mechanism of action, antimicrobial formulations should be tested following the synthesis immediately and after aging to ensure the long-term effectiveness of these materials in preventing DS.

Finally, one of the most important aspects to be considered in future investigations is to test these materials in a more reliable condition. Most of the reported studies were conducted in vitro against one fungal species, *C. albicans*. It will be more valuable to test these materials in a more challenging condition where the complexity of the oral cavity and the attachment of multi-species biofilms can be experienced [49,121,125]. Implementing in situ models, where the material can be tested inside the oral cavity, will provide more valuable information about the capabilities of such material to resist microbial attachment. Such a model will allow the designed materials to be challenged not only against *C. albicans* but also against the entire microbial communities inside the oral cavity where *C. albicans* can interact with other species.

In conclusion, there are several promising compounds that can be used to minimize microbial attachment and prevent the onset of DS. However, the use of these compounds requires further characterization and assessment, especially in the long-term evaluation. Besides, testing these compounds in a clinical translational model will offer a more comprehensive idea concerning the clinical reliability of such material. 

## Figures and Tables

**Table 1:** Summary of the bioactive agents incorporated into denture based resin (DBR) materials to minimize the growth of denture stomatitis-related pathogens.

Type of the Material		Main Findings
**Organic Compounds**	Henna	DBRs containing 5,7.5, and 10 wt.% of Henna achieved 1 to 2.5-log reduction against *C. albicans* [62] DBRs containing 0.5 wt.% of white and natural Henna inhibited the *C. albicans* growth without compromising the mechanical and physical properties of the material [63]
	Phytoncide Microcapsules	DBRs containing 1.25, 2.5, 3.75, and 5 wt.% of phytoncide demonstrated excellent biocompatibility and fungal reduction against *C. albicans*. However, the 5% concentration was associated with compromised mechanical properties [64] DBR containing 6 wt.% of type A and 15 wt.% of type B phytoncide microcapsules revealed a significant reduction against *C. albicans* growth [65], but the mechanical and physical properties were negatively affected [66]
	Neem	Incorporating 2.5 wt.% of neem was associated with around 2-log reduction against *C. albicans* growth over the neem-containing DBR samples [67]
	1,4-diazabicyclo[2.2.2]octane (DABCO) derivatives	One of the DABCO derivatives was found effective in inhibiting *C. albicans* when it was incorporated into DBRs [68]
**Inorganic Particles**	Silver	DBRs containing 0.1, 0.2, and 0.3 wt.% of silver demonstrated a significant reduction against *C. albicans* before and after one week of aging without compromising the mechanical properties and the polymerization kinetics of the material [69] DBRs containing 10 wt.% of silver vanadate nanoparticles [70] and 5 wt.% of silver nanoparticles [71] significantly eradicated the *C. albicans* growth Incorporating 0.5 to 1.5 wt.% of silver nanoparticles into DBRs reduced the *C. albicans* biofilm by 1 to 1.5-log
	Pre-reacted Glass Ionomer Fillers	Incorporating 5, 10, and 20 wt.% of pre-reacted glass ionomer fillers into DBRs reduced the attachment of *C. albicans* [72]
	Zinc oxide (ZnO)	Silanized ZnO nanoparticles (2.5, and 5 wt.%) demonstrated higher flexural strength values and potent antifungal action, up to 2-log reduction, against *C. albicans* [73]
	Zirconium dioxide (ZrO_2_)	ZrO_2_-containing DBRs inhibited the *C. albicans* biofilm growth by 0.5 to 1-log with improved flexural strength [74] At 1, 2.5, and 5 wt.%, ZrO_2_-containing DBRs achieved 0.5 to 2-log reduction against *C. albicans* biofilms before and after thermocycling [75]
	Silicon dioxide (SiO_2_NPs)	A significant reduction against *C. albicans* biofilms was observed with increasing the concentration of SiO_2_NPs, without compromising the mechanical properties when small concentrations were used [76]
	Titanium dioxide nanoparticles (TiO_2_NPs)	Incorporating TiO_2_NPs into DBRs at 1 and 2.5 wt.% reduced the growth of C. albicans by 0.5 to 1-log [77]
	Nanodiamonds (NDs)	Adding 0.25, 0.5, and 1 wt.% of nanodiamonds to DBRs inhibited the *C. albicans* growth by 1-log without compromising the mechanical properties [78,79]
**Polymeric Compounds**	Chitosan	Incorporating chitosan at 0.5–3 wt.% into DBRs was effective in inhibiting the growth of *C. albicans* [80]
	Fluoroalkyl acrylate	DBRs containing fluoroalkyl acrylate were able to reduce the attachment of *C. albicans* [81]
	2-hydroxyethyl methacrylate ester	Significant reduction against *C. albicans* adhesion was observed [82]
	Zirconium methacrylate (ZM)	DBRs containing ZM, TM, and DNBMT compounds demonstrated higher antifungal reduction against *C. albicans* and lower roughness than the control group [83]
	Tin methacrylate (TM)	
	Di-n-butyldimethacrylate-tin (DNBMT)	
	Poly (2-tert-butylaminoethyl) methacrylate (PTBAEMA)	Incorporating 10 wt.% of PTBAEMA into DBRs was effective to inhibit the growth of different bacterial species but not *C. albicans* [84]
	Phosphated poly(methyl methacrylate)	Incorporating 15 wt.% of phosphated poly(methyl methacrylate) into DBRs significantly reduced the attachment of *C. albicans* [85]
**Quaternary Ammonium Compounds**	Dimethylaminohexadecyl methacrylate (DMAHDM)	Incorporating DMAHDM at 3 wt.% into DBR material reduced the growth of *C. albicans* by 1-log. More reduction was observed when DMAHDM was combined with a protein-repellent agent named 2-methacryloyloxyethyl phosphorylcholine (MPC) [86]
	Dimethylaminododecyl methacrylate (DMADDM)	Incorporating 5, 10, and 20 wt.% of DMADDM into DBRs significantly reduced the metabolic activities and biofilm growth of *C. albicans* [87]
	N,N-dimethylaminoethyl methacrylate (DMAEMA)	DBRs containing DMAEMA reduced the *C. albicans* growth by 2-log. However, the mechanical properties, such as flexural strength and water sorption, were severely compromised [88]
**Protein-repelling Agents**	2-methacryloyloxyethyl phosphorylcholine (MPC)	When it was combined with DMAHDM, MPC-containing DBRs significantly reduced the growth of *C. albicans* [86]
**Antifungal medicaments**	Chlorohexidine	Adding fluconazole to DBRs did not affect the fracture toughness of the material, whereas Chlorohexidine addition negatively affected the fracture toughness [89]. Incorporating 10% of chlorhexidine into DBRs was found to inhibit the growth of *C. albicans* biofilms significantly [90] A larger inhibition zone was observed around DBR samples containing chlorhexidine compared to the control [91]

## Data Availability

Not applicable.
